# Patients’ and clinicians’ experiences of the DSM-5 Cultural Formulation Interview: A mixed method study in a Swedish outpatient setting

**DOI:** 10.1177/1363461520938917

**Published:** 2020-07-09

**Authors:** Malin Idar Wallin, Marie Dahlin, Lauri Nevonen, Sofie Bäärnhielm

**Affiliations:** 1 Karolinska Institutet; 2 Örebro University and Praktikertjänst Psychiatry

**Keywords:** clinical assessment, cultural formulation, cultural psychiatry, ethnicity and health

## Abstract

This study is an evaluation of clinicians’ and patients’ experiences of the core Cultural Formulation Interview (CFI) in DSM-5. The CFI provides a framework for gathering culturally relevant information, but its final form has not been sufficiently evaluated. Aims were to assess the Clinical Utility (CU), Feasibility (F) and Acceptability (A) of the CFI for clinicians and patients, and to explore clinicians’ experiences of using the CFI in a multicultural clinical setting in Sweden. A mixed-method design was applied, using the CFI Debriefing Instrument for Clinicians (*N* = 15) and a revised version of the Debriefing Instrument for Patients (*N* = 114) (DIC and DIP, scored from −2 to 2). Focus group interviews were conducted with clinicians. For patients (response rate 50%), the CU mean was 0.98 (*SD* = 0.93) and F mean 1.07 (*SD* = 0.83). Overall rating of the interview was 8.30 (*SD* = 1.75) on a scale from 0 and 10. For clinicians (response rate 94%), the CU mean was 1.14 (*SD* = 0.52), F 0.58 (*SD* = 0.93) and A 1.42 (*SD* = 0.44). From clinician focus-group interviews, the following themes were identified: approaching the patient and the problem in a new manner; co-creating rapport and understanding; and affecting clinical reasoning and assessment. Patients and clinicians found the CFI in DSM-5 to be a feasible, acceptable, and clinically useful assessment tool. The focus group interviews suggested that using the CFI at initial contact can help make psychiatric assessment patient-centred by facilitating patients’ illness narratives. We argue for further refinements of the CFI.

## Introduction

Due to globalization and migration, there is an increasing need for culturally sensitive assessment tools in mental health care. Several studies show a healthy-migrant effect in some countries, in that immigrants are on average healthier than members of the native-born population (Kennedy, Kidd, McDonald, & Biddle, 2014; Rivera, Casal, & Currais, 2016). However, there are also studies indicating that immigrants in general, and refugees in particular, are at greater risk of mental disorders (Fazel, Wheeler, & Danesh, 2005; Hollander, Bruce, Burstrom, & Ekblad, 2013; Hollander et al., 2016), and that elevated risk also affects the second generation of immigrants (Bourque, van der Ven, & Malla, 2011). In Sweden, over the past few decades, it has been possible to identify waves of refugee migration from conflict areas (Swedish Migration Agency, 2019). European studies, however, show a lower utilization of psychiatric inpatient and outpatient care among immigrants, refugees included (Lindert, Schouler-Ocak, Heinz, & Priebe, 2008; Priebe, Giacco, & El-Nagib, 2016). Variation in the expression and understanding of symptoms of psychiatric disorders may pose difficulties in transcultural psychiatric diagnosis (American Psychiatric Association, 2013), especially in the cases of newly-arrived immigrants and refugees (Adeponle, Thombs, Groleau, Jarvis, & Kirmayer, 2012). Diagnostic difficulties increase the risk that mental disorders among immigrants and refugees go undetected, or are misdiagnosed. Incorrect diagnoses can lead to poor adherence to treatment, sub-optimal treatment, and, ultimately, even a lack of treatment.

### DSM-5 Cultural Formulation Interview

To enhance awareness of culture and context in applying psychiatric diagnoses, DSM-5, the Diagnostic and Statistical Manual of Mental Disorders of the American Psychiatric Association (2013) includes a Cultural Formulation Interview (CFI) (Section III, pp. 749–759). The CFI provides a framework for gathering culturally sensitive information, enabling clinicians to identify cultural and contextual factors of relevance to diagnosis and treatment. It may also be helpful in judging illness severity, in understanding disagreements between patient and clinician, and in situations of limited commitment and adherence to treatment (Diagnostic and Statistical Manual of Mental Disorders: DSM-5, 2013). Additionally, the CFI can improve patient-clinician communication by increasing rapport (Aggarwal, Desilva, Nicasio, Boiler, & Lewis-Fernández, 2015, Muralidharan et al., 2017), be useful in creating trust, elicit important non-diagnostic contextual information, and support treatment planning (Ramirez Stege & Yarris, 2017). The CFI does not replace traditional diagnostic tools and skills, but is an additional instrument for co-ordinating and mapping information (Bäärnhielm, Rohlof, Misiani, Mutiso, & Mwangi, 2016).

The operationalization of the CFI in DSM-5 was based on research with previous regional interview guides, developed in different countries and languages. The points of departure were the Outline for Cultural Formulation (OCF) in DSM-IV (Lewis-Fernández et al., 2014) and an international field trial testing a pilot version of the CFI for DSM-5 (Lewis-Fernández et al., 2017).

The CFI calls for systematic assessment of cultural and contextual factors related to four domains: 1) definition of the problem, 2) perception of cause, context, and support, 3) self-coping and past help seeking, and 4) current help seeking. The CFI can be used for the initial assessment of all patients, and may be especially helpful in transcultural diagnostic processes (Diagnostic and Statistical Manual of Mental Disorders: DSM-5, 2013). The core CFI with 16 questions can be supplemented, if needed, by an informant version and by questions from 12 supplementary modules supporting further information-gathering (Diagnostic and Statistical Manual of Mental Disorders: DSM-5, 2013).

Using the CFI is a patient-centred method to support the exploration of the impact of culture and context in an individualized and non-stereotypic way. The value of culturally sensitive diagnostic methods has been shown in earlier studies evaluating the OCF in DSM-IV (Adeponle et al., 2012; Bäärnhielm, Aberg Wistedt, & Rosso, 2015; Groen, Richters, Laban, & Deville, 2017; Rohlof, 2018; Scarpinati Rosso & Bäärnhielm, 2012).

Before the CFI was included in DSM-5, its Clinical Utility, Feasibility and Acceptability were evaluated in a multi-centre field trial (Aggarwal et al., 2015; Diaz, Anez, Silva, Paris, & Davidson, 2017; Lewis-Fernández et al., 2017; Paralikar, Sarmukaddam, Patil, Nulkar, & Weiss, 2015; Rohlof, 2018). The results show that both patients and clinicians found the tested four-item pilot version of the core CFI to be acceptable, feasible, and clinically useful (Lewis-Fernández et al., 2017). As a part of the field trial, one study evaluated relatives’ views on its utility, feasibility, and acceptability, showing that they had a positive view on the CFI. These findings were supported by results from qualitative analyses of debriefing interviews (Hinton et al., 2015).

The results of the DSM field trial studies revealed differences between centres, necessitating local evaluations. Further, in the DSM-5 field trial, clinicians had only used the CFI on a limited number of occasions, which is why an evaluation of the perceptions of more experienced CFI users may add valuable knowledge. Despite the DSM-5 field trial, which indicated that the pilot version of the CFI in DSM-5 was a useful tool, few studies have evaluated the final version of the CFI that is included in DSM-5. In a qualitative study, in a Mexican setting, the CFI was found to be a way of building trust and increasing providers’ understanding of contextual factors influencing mental illness (Ramirez Stege & Yarris, 2017). In another qualitative study, in the United States, using the CFI on patients with psychotic disorders, the CFI was reported to enhance patient and clinician rapport and to be a way of obtaining meaningful health narratives (Muralidharan et al., 2017).

The present study aimed to assess the clinical utility, feasibility and acceptability of the core CFI in DSM-5 for both clinicians and patients, and to explore clinicians’ experiences of using the core CFI in an outpatient clinical setting in Stockholm, Sweden.

## Methods

### Study design and setting

The present study was conducted from August 2015 to May 2017. Data were collected from the intervention group in a clinical RCT comparing CFI and non-CFI clinical assessments. We collected data from the clinicians (trained in using the CFI) and from patients in the CFI intervention group. A mixed-method approach was chosen, using questionnaires and focus group interviews with the clinicians.

The study was conducted at an outpatient psychiatric facility, Praktikertjänst Psychiatry AB in Stockholm Sweden. In 2017, its Järva Clinic had 114 full-time employees, including psychiatrists, psychiatric nurses, psychologists, clinical social-workers, and caretakers, at three different geographical sites, and treated 4,038 individual patients. The catchment area in western Stockholm has a very mixed population, with suburbs that are home to many immigrants and refugees. The proportion of patients with an immigrant background (first and second generation) is 75%. Many have a refugee background, and most of the immigrant patients come from Asia (e.g., Turkey, Iran, Iraq and Syria) and Africa (e.g., Somalia and Eritrea). The socio-economic status of the population is precarious, and the occurrence of physical and mental health problems is high. The most common activities undertaken at the clinic are: outreach programs, investigations, various sorts of psychotherapy, psychopharmacological treatment, socialization groups, home visits, basic somatic examinations, prescriptions, referrals and collaboration with social services. The clinic has employees who master a total of 23 different languages between them, but it employs interpreters when needed.

In the study, the core CFI interview, without any supplement, was included in the standard psychiatric diagnostic procedure, which, in Stockholm County Council, contains a clinical diagnostic interview. Additionally, several web-based screening tools^1^ were used. Although clinicians from different professions may perform the standardized psychiatric assessments and treatment planning, a psychologist or psychiatrist is responsible for making the formal diagnostic categorization according to ICD-10. This categorization should be based on all information gathered in the standard diagnostic procedure.

### Sample

#### Patients

The CFI was applied in consultations with 114 patients who had not been in contact with psychiatric care during the preceding two years. Accordingly, the clinicians and the patients had no previous contact. Forty-two patients were known to be non-Swedish born, with their origins in the Middle East (*n* = 19), Europe (*n* = 11), Asia (*n* = 4), South America (*n* = 4), and Africa (*n* = 4), while the origins of 14 patients were unknown. Most had lived in Sweden for more than 10 years. Some patients had been referred to the clinic, mainly from primary care clinics, while some came on their own initiative (25%). All were included as participants in the current study.

#### Clinicians

The clinicians performing the CFI were psychologists, psychiatric nurses, counsellors, and psychotherapists of diverse cultural backgrounds. According to routine in clinical settings, these groups of professionals collect initial data for diagnostic evaluation. Based on these data, a final nosological evaluation to formulate an ICD diagnostic label in the medical record is made by a psychologist or psychiatrist. All the clinicians performing the CFI had significant experience of working in a multi-cultural location, but no previous experience of using the CFI. On starting in the study, they participated in two half-day training courses, including lectures and roleplay using the CFI and discussions based on their own clinical cases. The training was led by the last author (SB), who was also a member of the DSM-5 Cross Cultural Issues Subgroup (DCCIS) (Lewis-Fernández et al., 2014). The number of CFIs performed in the study per clinician ranged between one and 30.

### Measures

To assess perception of the CFI, we used the Debriefing Instrument for Patients (DIP) and for Clinicians (DIC). Both instruments were designed for the DSM-5 CFI field trial to measure three factors: Clinical Utility, Feasibility, and Acceptability (Lewis-Fernández et al., 2017). Swedish translations of the DIP were made within our group. SB translated from English to Swedish, after which the translations were back-translated into English by another team member, and the versions discussed and processed. Official translators made translations of the DIP into other relevant languages, which were then checked by bilingual clinicians. The languages were: Arabic, Bosnian, Eritrean, Farsi, Finnish, Kurmanji (north Kurdish), Polish, Russian, Somali, Sorani (south Kurdish), Spanish and Turkish.

Due to psychometric concerns about the DIP that were raised in the field study, we reached an agreement with its authors to revise the instrument by removing items that were difficult to understand and translate. Items reported to be problematic were the two negatively worded items: ‘Took more time to share my perspective than I wanted’ in the case of Feasibility, and ‘Were too personal’ in the case of Acceptability. We did not want to exclude these items, which were considered relevant to patients; and, during the decision process, we chose to prioritize aspects of face validity and assumptions regarding patient relevance. All items were discussed with clinicians from the catchment area. This process resulted in several changes: (1) the omission of three items from the original Clinical Utility factor: Item 5 (‘Gave me confidence that the clinician understood my situation’), item 6 (‘Helped me identify things that could get in the way of my treatment’), and Item.8 (‘Were useful overall’). (See Table 1 for the remaining items); (2) The omission of one item from Feasibility factor: no. 11 (‘Improved the flow of the interview’), which was considered difficult to translate and make comprehensible in Swedish (See Table 1 for the remaining items); (3) The complete change of the Acceptability factor, into a one-item question (‘How did you perceive the questions overall?’) rated on a visual analogue scale (VAS) ranging from 1(‘Too personal’), to 10 (‘Good, helped me tell my story’).

**Table 1.** Patients’ ratings of the revised Debriefing Instrument for Patients (DIP), *n* = 57.

Factor	Item	Item mean (*SD*)	Factor mean (*SD*)
Clinical utility	Helped me explain my main concerns	1.09 (0.93)	0.98 (0.93)
	Helped me communicate important aspects of my background, such as religious faith and/or culture	1.01 (1.06)	
	Helped me understand how my background and current situation affect my problem	0.70 (1.32)	
	Helped me explain what kind of help I would like	1.02 (1.10)	
	Encouraged me to share important information that I might not have mentioned otherwise	1.09 (1.17)	
Feasibility	Were easy to understand	1.04 (0.94)	1.07 (0.83)
	Took more time to share my perspective than I wanted (R)	1.11 (1.03)	
Acceptability	Overall perception of questions	8.30 (1.75)	–

*SD* = Standard Deviation.

R = Reversed.

All items (apart from DIP/Acceptability) in the DIP and DIC had five response alternatives. Scoring followed the previous study (Lewis-Fernández et al., 2017), with 2 (Strongly Agree) and 1 (Agree) for positive responses, and -1 (Disagree) and -2 (Strongly Disagree) for negative responses. In addition, patients were asked to state (yes/no) whether any of the questions made them feel uncomfortable.

No revisions were made to the DIC, which has questions for the scoring of Clinical Utility, Feasibility, and Acceptability. The DIC also includes three open-ended questions: ‘Which of the CFI questions were the most useful? Why?’, ‘Which were least useful? Why?’, and ‘What would the challenges be to incorporate the CFI questions into your routine clinical practice?’ Additionally, the DIC includes questions where the clinician is asked to rate whether the written CFI guidelines, ‘… were clear and easy to understand’ and ‘… prepared me well to administer the CFI questions’ (response alternatives and scoring as above).

### Procedure

Patients were invited to take part in the study during their initial meeting with a clinician. Patients who agreed to participate received the DIP questionnaire (*N* = 114) upon completion of the CFI interview. They had the option to complete this privately or with assistance from the clinician or an interpreter. They were able to return the questionnaire to the clinician or anonymously in a box at the clinic reception. The questionnaire was translated and available in 12 languages. An interpreter was used in consultations with 22 patients. They did not reveal their identity or provide any baseline data on the form. All clinicians (*N* = 15) received their questionnaire after all their interviews had been performed, i.e., upon completion of the RCT intervention.

Two focus group interviews were conducted with clinicians at the end of the period for data collection, after they had used the CFI on a regular basis for two years. The interviews took place at two of the three clinics and lasted approximately 90 minutes. The interviews were audio-recorded and transcribed verbatim. The first author (MIW) conducted the interviews in the presence of an observer; a semi-structured interview guide was used for the interviews (Appendix 1). Four clinicians were no longer employed at the clinic by the time of the focus group interviews, and could not be reached, leaving 11 eligible participants. One potential participant in each focus group was absent from work at time of interview resulting in nine actual participants, five in one group and four in the other.

### Data analysis

#### Statistical analyses

There were 3.8% item records missing for the DIP and 2.6% missing for the DIC. Missing data were imputed using the mean of the remaining items within each factor. There were no instances of more than one missing item score per factor. The negatively worded item ‘Took more time to share my perspective than I wanted’ was scored in reverse. We computed means and standard deviations for each item in both instruments, as well as for the factors Clinical Utility and Feasibility for both instruments, and Acceptability for the DIC. The open-ended questions in the DIC questionnaire were analysed and included in the results from the focus group interviews. Where applicable, we assessed internal consistency by means of Cronbach’s alpha for the DIP Clinical Utility factor and for all the DIC factors. Cronbach’s alpha for Clinical Utility was 0.88 in the case of the DIP (five items). For the DIC, internal consistency was 0.82 for Clinical Utility (10 items) and 0.82 for Feasibility (four items). Due to the very low internal consistency of the DIC Acceptability factor (0.16, four items), we excluded item 15 (‘Helped make the patient feel more at ease during the interview’), resulting in a factor of three items with a still-low internal consistency of 0.55.

#### Qualitative analysis

Qualitative data from the clinicians consisted of two transcribed focus group interviews and answers to the open questions in the DIC questionnaires. The interviews and the open-ended questions in the DIC questionnaire were analysed for themes using qualitative content analysis (Rivas, 2012), with support from the software program NVivo11 (2015). We first indexed the data descriptively in terms of content, bringing together all data belonging to diverse index areas. Three index areas were identified: Experiences from using the CFI, Clinical implementation, and Comments on the wording of the CFI questions. For further analysis of the different index areas, the following procedure was adopted. The first step in organizing the text was to identify meaning units that were relevant to the information from the interviews. Second, codes were given to the meaning units that were representative of the content of each unit. Third, the codes were grouped into preliminary sub-themes, based on common content. Fourth, broader themes were identified describing the sub-theme content. Finally, each sub-theme was explored further, and the themes were refined. Thirteen sub-themes and three overarching themes related to the clinicians**’** experiences were identified. The analytic process is illustrated in Table 2.

**Table 2.** Exemplifying the coding process.

Meaning units	Codes	Sub-theme	Theme
“You reach an understanding of their view of the situation, you don’t start out from your usual models and assumptions.”“That you get information, but maybe you ask more before you make a diagnostic decision. That it takes more … But it is a piece in the puzzle, definitely.” “Yes, like you say, they are empowered by this.”	An understanding of the patient’s pictureA piece in the diagnostic puzzleThey are empowered	A revised understandingDetecting diagnostic cluesEmpowering the patient	Co-creating an understandingAffecting clinical reasoning and assessmentValue for the patient

### Ethical considerations

Oral and written information for patients, translated into 12 languages, was given, underlining that participation was voluntary and could be withdrawn at any time without negative consequences. Ethical approval for the study was obtained from the Regional Ethical Review Board in Stockholm (2015/243-31/2).

## Results

### Patient questionnaires

Among the patients, 57 out of a total of 114 (50%) returned their questionnaires. In a few instances (exact numbers not known), the patient did not receive a questionnaire due to clinicians’ lack of time. All responders used the Swedish DIP version. The DIP results are presented in Table 1. The Clinical Utility mean was 0.98 (*SD* = 0.93) with individual items ranging between 0.70 and 1.09. The Feasibility mean was 1.07 (*SD* = 0.83). Overall perception (rating) of the questions in the interview had a mean of 8.30 (*SD* = 1.75) on the VAS (between 0 and 10). Eight patients, 14.5%, reported that they perceived some questions in the interview as discomforting. Among the 57 patients, six patients responded to the open question regarding how they were troubled or made to feel uncomfortable; the following answers were given: “(It was) generally uncomfortable to talk about.”; “(It was) painful to realize that the cause of my problems has to do with me.”; “I had trouble understanding what the question about identity meant.”; “The question about what kind of help I want was difficult to answer – don’t know.”; “Difficult to explain question number four (Why do you think this is happening to you? What do you think are the causes of your problem?).”; “The question about culture and background. I am afraid that other people will think badly about me because I have become rather Swedish.”

#### Clinician questionnaires

All 15 clinicians responded to the DIC (Table 3). The Clinical Utility factor mean was 1.14 (*SD* = 0.52). Further, all the individual items were positively rated: from ‘Gave me confidence in the diagnosis’ (*M* = 0.64; *SD* = 0.87) to ‘Helped me identify additional aspects or dimensions of the patient’s clinical problems’ (M = 1.47; *SD* = 0.52). The Feasibility factor mean was lower (*M* = 0.58; *SD* = 0.93), and one item was negatively rated, ‘Were easily understood by the patient’ (*M* = −0.07; *SD* = 1.22). Acceptability was rated the highest, (*M* = 1.42; *SD* = 0.44); mean scores among the items on the factor ranged from *M* = 1.20; *SD* = 0.77 to *M* = 1.53; *SD* = 0.52 for both ‘Facilitated a good assessment of cultural factors relevant to clinical care’ and ‘I would recommend for use by other mental health clinicians’. The mean of item 15, ‘Helped make the patient feel more at ease during the interview’ (which was excluded from the Acceptability factor as a result of the internal consistency analysis) was lower than that of the items it contained, *M* = 1.00 (*SD* = 0.96). Two additional questions about the instructions for the interview had positive ratings (*M* = 1.21–1.31; *SD* = 0.43–0.48).

**Table 3.** Clinicians’ ratings on the Debriefing Instrument for Clinicians (DIC), N = 15.

Factor	Item	Item mean (SD)	Factor mean (SD)
Clinical Utility	Helped me understand the patient’s cultural background	1.33 (0.49)	1.14 (0.52)
	Clarified the patient's ideas about the causes of the problem	1.13 (0.99)	
	Clarified my understanding of the patient’s symptoms and problems	0.93 (0.88)	
	Gave me confidence in the diagnosis	0.64 (0.87)	
	Facilitated treatment planning and implementation	1.10 (0.97)	
	Helped me identify issues that could interfere with treatment adherence	1.27 (1.03)	
	Helped me identify additional aspects or dimensions of the patient's clinical problems	1.47 (0.52)	
	Helped me assess the severity of the patient’s problems	1.00 (0.93)	
	Facilitated my rapport with the patient	1.10 (0.97)	
	Were useful overall	1.40 (0.51)	
Feasibility	Were easy to administer	0.60 (1.06)	0.58 (0.93)
	Were easily understood by the patient	−0.07 (1.22)	
	Contributed positively to the flow of my clinical interview	0.73 (1.33)	
	Could be integrated easily into my routine clinical practice	1.07 (0.96)	
Acceptability	Helped make the patient feel more at ease during the interview	1.00 (0.96)	1.42 (0.44)
	Can be incorporated by mental health clinicians into routine clinical interviews	1.20 (0.77)	
	Facilitated a good assessment of cultural factors relevant to clinical care	1.53 (0.52)	
	I would recommend for use by other mental health clinicians	1.53 (0.52)	
The instructions for the interview	… were clear and easy to understand	1.31 (0.48)	
	… prepared me well to administer the CFI questions	1.21 (0.43)	

*SD* = Standard Deviation.

CFI = Cultural Formulation Interview.

### Focus group interviews and responses to open questions

Clinicians talked about their experiences of using the CFI in comparison with their ordinary clinical procedure. From the analysis of the clinicians’ experiences of using the CFI, the following themes were retrieved: approaching the patient and the problem in a new manner; co-creating rapport and understanding; and, affecting clinical reasoning and assessment. The themes and sub-themes are presented in Table 4 and described in the text that follows.

**Table 4.** Themes and subthemes from the qualitative data.

Themes	Subthemes	Focus group mentions
Approaching the patient and the problem in a new manner	Different questions	19
	Exploring the problem together	19
	Facilitating the patient’s narrative	12
	Illuminating the patient’s resources	5
Co-creating rapport and understanding	Building rapport	7
	Providing a richer picture of the patient	23
	Contextualizing the person and the problem	10
	Reaching a revised understanding	16
	Empowering the patient	12
Affecting clinical reasoning and assessment	Detecting diagnostic clues	8
	Effects on choice of treatment	13
	Facilitating the patient’s contribution	36
	An echo of the patient’s voice	9

#### Approaching the patient and the problem in a new manner

The first theme focuses on how the clinicians approach the patient in a new way when using the CFI compared with the ordinary procedure. The theme includes the following sub-themes: different questions; exploring the problem together; facilitating the patient’s narrative; and, illuminating the patient’s resources.

The informants described how using the CFI entailed asking questions different to those in their ordinary procedure, and how they needed to abandon their original assumptions. They found the CFI particularly helpful when talking about delicate matters, such as religiosity and sexual orientation or, for some patients, just talking about having mental health problems, and also for eliciting new and unexpected information. The clinicians reported that the CFI questions about identity and cultural background were ones that the patients had not been asked before. They described the CFI as valuable in initial clinical assessment; for example:

“Sometimes they say good that you ask, nobody else has asked this, they say” (referring to cultural identity and background).

“It [the CFI] is quite broad and covers a lot of questions I would like to ask when I formulate a patient’s case.”

The clinicians reported that the CFI interview supported them in approaching the patient and the problem with curiosity, as part of a mutual exploration of the problem. The CFI questions created a sense of working together and engaged the clinicians in an enhanced listening mode, since they were not confined to ordinary checklists and templates. Examples were:

“You explore together, instead of labelling the patient. Because that might silence the patient; this, instead, opens things up in a way.”

“… that you are not so bound to these interview templates and the like; here … you simply need to step back and listen more to the individual.”

According to the clinicians, the CFI allowed the patient’s narrative to play a central role in the clinical situation. It generated subtleties in the information gathered and elicited new dimensions of the patient and the problem, while also accessing patients’ views on their daily life. The clinicians found this to result in a more complex picture, enhancing their understanding of the problem and the person. Examples included:

“It results in another description, another picture of their daily life” (compared to their ordinary procedures).

“There are subtleties somehow; it’s the individual’s narrative, not my templates that I check if the patient has this and this.”

The CFI helped shed light on the patient’s resources, such as previous coping strategies, family members’ roles, and how the existing or lack of resources contributed to, or interfered with, treatment and care. Examples:

“What they themselves have … had as their coping strategies. Where you sometimes also find … they were on the right track and then I have tried to support those … bits.”

“It becomes more like having a full picture of how this person lives, what social resources there are, how this person thinks.”

#### Co-creating rapport and understanding

The second theme focused on how the CFI started a process of co-creating better rapport, in cooperation with the patient, and a revised and shared understanding of the individual and the problem. Information on the patient’s perspective created a richer picture for the clinicians. They described how the CFI questions had an empowering effect on patients by making them feel acknowledged and listened to. The theme includes the sub-themes: building rapport; a richer picture of the patient; contextualizing the person and the problem; a revised understanding; and, empowering the patient.

The CFI was found to support a good working alliance, where the open questions allowed the patient’s view to emerge, and enabled a positive alliance-building atmosphere to be created. For clinicians, abandoning the mandatory structure made for more of a listening approach that contributed to good rapport with the patient.

“It is like … perhaps it becomes a better atmosphere than when you … pressurise the patient too much. It [the CFI] allows their own words to come through in a way.”

“I think that one of the patients I interviewed, he… he thought it was a bit … well – interesting. He was kind of happy to take part. After that, when I see him, I feel that our clinician–patient alliance has become very good.”

The clinicians also emphasised the need to adjust the wording of the CFI questions and the time needed for the CFI according to the requirements of the patient.

The CFI helped clinicians to obtain a more holistic perspective. A clinician’s narrow focus on the patient’s psychiatric diagnostic label could be broadened when a more comprehensive picture of the person and the context of the problem emerged. As one participant stated: “It [the CFI] gives a much richer picture.”

Family members’ perceptions of the problem, and the traditions and beliefs that sometimes affect the patient and treatment adherence, were revealed by the CFI. It was found useful when exploring the patient’s contextual situation, even with patients without an immigrant background. The clinicians did not refer to background and identity as closely related to ethnicity, and made a point of ethnicity not being the most important factor in information related to background and identity.

The clinicians also saw the CFI questions as providing a good opportunity for the patients to formulate their reflections, helping them to place themselves and their problem into a context; for example:

“What context are they living in, what is important to their identity, or how might it be related to cultural factors. What particularly complicates their receiving of help or their being understood and seen.”

The CFI helped clinicians and patients to reach a revised understanding of the problem, including the need for help. For example, in the case of a patient exposed to a great deal of violence while growing up, the CFI questions helped the clinician to get a more nuanced understanding of the patient and the patient’s problem. Examples included:

“You get their picture of how they understand … causes and how they view matters … if they even see a connection with their identity and background, if they have thought of it like that. Yes, that’s a good thing, I think.”

“… it’s more like you increase your understanding in a way. A bit more … a deepened understanding. A bit more … how this person looks at it.”

The CFI was regarded as contributing to making patients feel important; the clinicians perceived the patients’ as experiencing being listened to, having their contributions regarded as valuable and important, and that they were “empowered”. As one participant put it, the CFI gave: “…a sense that the patients feel that they are seen; they are interviewed, they are in … a context.”

#### Affecting clinical reasoning and assessment

The third theme concerns how the CFI questions impact clinical reasoning and assessment. It focuses on how the CFI contributes diagnostic clues and has an impact on choice of treatment. The clinicians said that using the CFI seemed to make the patient’s voice echo in the medical records. The sub-themes are: detecting diagnostic clues; effects on choice of treatment; facilitating the patient’s contribution; and an echo of the patient’s voice.

The information gained through the CFI gave the clinicians diagnostic clues and helped them identify which aspects of the problem were related to psychiatric illness. Additionally, it contributed to knowledge about problematic aspects in the patient’s background, clarifying what the clinicians should examine more closely. Examples:

“I guess it has also sometimes … well become clear that it is not psychiatric care they need, but in fact other things that … that have a greater influence on their ill health.”

“That you get information, but maybe you ask more before you make a diagnostic decision. That it takes more… But it is a piece in the puzzle, definitely.”

Answers to the CFI questions supported clinicians in trying to identify the most appropriate treatment and prognoses for treatment adherence. The answers also revealed the patient’s ability to reflect and provide information about coping strategies from the past – useful in further treatment planning and care. Examples:

“These challenges they have managed before, that will… Then there is good hope that this will actually work, despite the complex situation.”

“If they say they only believe in pharmacological treatment then … then I will not put in two years of therapy so to say.”

The clinicians found that the CFI supported the patient’s contribution, making the clinical assessment more patient-centred. The open CFI questions moved clinical conversations to a more in-depth level where patients were supported in talking about their problems; for example: “Sometimes when you ask these concrete questions they have the courage to talk about a problem that they may never have told anybody about.” One clinician referred to a patient who said: “… but now when you ask this question, I have the courage to speak out, and that was … It has helped me to manage my illness.”

Documentation from the CFI in the medical records was evident in quotes of the patient’s own words, thereby leaving an echo of the patient’s voice, unlike in the usual documentation of a diagnostic procedure. Examples include: “And it is the patient’s words and narratives I should write down, not my assessments,” and “No, you have to write down their answers, you cannot kind of reinterpret them.”

#### Comments on the wording of CFI questions

The clinicians had diverse views on the wordings of the CFI questions, particularly the question that included both identity and background. Some clinicians thought the phrasing of the questions was abstract and complicated for some patients, especially those with a low intellectual capacity or low educational level and psychotic patients. In consultations involving an interpreter, the clinicians found the CFI and the support text explaining the questions to be too complicated. For example: “All the questions are useful. Some of them are difficult for the patient to understand, the one about cultural identity for example, but they are good questions” (quote from a questionnaire).

#### Comments on clinical implementation

The practical aspects of implementation and actual use of the CFI findings were discussed in the focus group interviews. The discussion took place in relation to the actual work situation, with a high influx of new patients and all the different measurements in the standardized initial assessment procedure. Examples were:

“Since it is such a structured interview, it has been helpful to kind of have something to lean on, I think.”

“Timewise it can be hard to fit it in.”

“You have more to document, so there is something else to make time for. But, apart from that, it is not difficult.”

While all clinicians participate in treatment planning, it is psychologists and psychiatrists who are responsible for making the formal diagnostic ICD-10 categorization. The participating clinicians were concerned that time constraints hindered the MDs in making full use of the CFI information in their diagnostic decisions. However, the interviewed clinicians used the information gathered in their treatment planning and recommended its use.

## Discussion

The study aimed to assess the Clinical Utility, Feasibility and Acceptability of the core CFI in DSM-5 for both clinicians and patients, and to explore clinicians’ experiences of using the CFI with new patients in a multicultural clinical setting in Sweden. The patient and clinician questionnaires, as well as focus group interviews with clinicians, show positive results for the Clinical Utility, Feasibility and Acceptability of the core CFI.

### Strengths and limitations

One strength of the study is its mixed method design, which provides us with both quantitative data on patients’ and clinicians’ views of the CFI in DSM-5 and in-depth information on the clinicians’ experiences of using the CFI questions in their clinical assessments. The study was conducted in a naturalistic setting, which has both limitations and strengths. Sampling and data collection, in some instances, suffered from shortcomings in rigour, which may have reduced reliability. However, validity is strengthened by the study having taken place in a busy clinical setting, with a very mixed multicultural population, including migrants, refugees, and people who were illiterate or who had language difficulties.

An interpreter was used with 22 of the patients in the study, which has both strengths and limitations. A strength is that translation gave the patients the opportunity to express themselves in their mother tongue. A limitation is that there were translation difficulties with some of the CFI questions. A different strength is that the clinicians were experienced in working in multicultural settings.

One significant limitation of this study is the low response rate from patients, which may have several explanations. Due to clinicians’ lack of time, the DIP questionnaire was not always administered to the patient after the CFI. Some patients received the questionnaire but did not return it, possibly due to concerns about anonymity or because responding to the questionnaire entailed having to rate their new clinical contact. It is also possible that they did not prioritize the completion of the DIP form because they were struggling to cope with initial contact with a new clinic and suffering from poor mental health. A limitation is that, due to the DIP anonymity, we cannot analyse the results in relation to social and cultural characteristics of the patients. We revised the DIP, which we believe increased comprehensibility of the instrument in the current Swedish context. But one obvious drawback of revision is that it reduced generalizability, including comparability with earlier studies. Further, the revision probably resulted in reduced variance.

The small sample size of 15 clinicians may be a limitation, but those involved were given the opportunity to use the CFI with many patients, thereby increasing their competence in using it. Some of the clinicians did not use the CFI more than one or a few times, and, for a few of them, it had been a long time since they had used the CFI when the focus group interview took place. However, most of the clinicians used the CFI for several patients as a part of their routine work, and the focus group interviews were conducted when the data collection for the study was nearly finalised. There were very few missing observations in either the DIC or the DIP, and in no instance was there more than one score missing within a single factor. The results from the clinician sample, consisting of only 15 subjects, however, was particularly vulnerable to missing data. Thus, in order not to lose all the data for any individual participant on any factor, we imputed missing data using the mean of the remaining item scores for each factor. Imputation may, in theory, have led to a loss of variance, but – given the limited number of missing observations – we do not think it has had any significant impact on our results or interpretations.

### Debriefing instrument for patients

The results indicate that the patients’ perceptions of the Clinical Utility, Feasibility and Acceptability of the CFI were mainly positive. As our sample is small and the response rate is low (50%), results should be interpreted with some caution. Importantly, we revised the questionnaire, which is why comparisons with earlier studies are not definitive and mainly serve purpose of orientation rather than strict comparison. We had removed three out of eight items from the Clinical Utility factor and one out of three from the Feasibility factor. The main reason for this was that we assumed an improvement in relevance and comprehensibility for the patient. There were very few missing records, which are not believed to have had an impact on results.

The mean for Feasibility (1.07) is slightly lower than in the international field trial of the pilot version of the CFI in DSM-5 (1.33) (Lewis-Fernández et al., 2017). The mean for Clinical Utility (0.98) is also lower than that in the field trial (1.26). The slightly lower means for Feasibility and Clinical Utility may be due to all patients in this study being new to the clinic, and to many of them having language difficulties. This means that that the patients may have had difficulties in fully understanding the CFI questions. For an interpreter, which was used in some cases, there were challenges in translating some of the questions. The patients answering the questionnaire responded positively to the question about overall perception of the CFI (mean of 8.30 out of 10). There were only a few comments on the open-ended questions about why the questions made the patients feel uncomfortable; there were a variety of reasons for this.

### Debriefing instrument for clinicians

The results for Clinical Utility, Feasibility and Acceptability of the CFI from the DIC were positive, except for the question, ‘Were easily understood by the patients’, which stood out as the only question with a negative response value. It might be that the translation from English to Swedish contributed to this, with the questions in Swedish being perceived as more abstract than the original questions in English. The clinical situation, with interpreters and several patients not having Swedish as their mother tongue, may also have contributed to language difficulties using the CFI. The results from the DIC tended to be more positive than those from the field trial. A possible explanation is that the clinicians in the current study had become more experienced in using the CFI since they had used it over a lengthy period of time, i.e., 1.5 years. They also had had lengthy experience of working in a multicultural clinical setting, which might have had an impact on the results for the highest rated DIC subdomain, Acceptability. These results may be generalizable to settings where clinicians have substantial experience of a multicultural setting and training in using the CFI. Although there were fewer clinicians than patients responding to the instruments, the standard deviations were generally smaller for the DIC than for the DIP, indicating that the clinicians are a small but homogenous group. While the clinicians seemed to think that the CFI questions were difficult for the patients to understand, the patients’ DIP results for ‘The questions were easy to understand’ were high.

### Findings from the focus group interviews

In the focus group interviews, the clinicians compared using the CFI with their ordinary procedures. The CFI compelled the clinicians to approach patients in a new manner, making them ask different questions and explore the problem together. The clinicians found that the new approach facilitated the patient’s narration and illuminated the patient’s resources. The clinicians described how the CFI contributed to co-creating a better rapport, in co-operation with the patient, creating a richer picture of the patient, and contextualizing the person and the problem. This resulted in mutual revised understanding and had an empowering effect on the patient. The information gathered from the CFI also affected clinical reasoning and assessment by providing the clinicians with diagnostic clues to choice of treatment. The CFI also facilitated the patient’s contribution and left an echo of the patient’s voice in the medical record. The results support using the CFI at initial contact, making psychiatric assessment more patient-centred by facilitating patients’ illness narratives.

Although the clinicians participating in the focus group reported positive experiences of using the CFI, they were concerned about clinical implementation, since they feared that time constraints might restrict the possibility of making full use of the CFI information in diagnostic decisions. This may explain why the Feasibility subdomain was rated lower than the other subdomains in the DIC. The present study found time constraints and difficulties in conveying the CFI findings to all the members of the clinical team to be major concerns regarding implementation. They may result in important information about the patient being lost in terms of clinical use.

Results from the focus group interviews were used to gain a deeper understanding of the results from the DIP and DIC questionnaires. The clinicians had a positive perception of the overall use of the CFI questions, which is confirmed by the DIC results. There was a consensus among the clinicians regarding their experiences of using the CFI and clinical implementation. However, there was variety in their comments concerning the wordings of the CFI questions.

The clinicians said that they would recommend the use of the CFI to other mental health care practitioners and regarded it as a useful tool for gathering valuable information at the initial clinical assessment. The DIC results and focus group interviews are consistent in this regard. The focus group interviews reveal that the clinicians found the interview questions to be difficult for some patients to understand, as reflected in the negative responses to the DIC item ‘Were easily understood by the patients’. Even so, overall, the clinicians found the CFI helpful with regard to understanding the patients’ problems, for themselves as well as for the patients. The clinicians said that the responses to the CFI questions brought out subtleties in the information gathered and influenced their clinical reasoning and assessment. This is confirmed by positive results for the item, ‘Helped me identify additional aspects or dimensions of the patient’s clinical problems’ from the DIC. The DIC results showed that the clinicians experienced the CFI questions as facilitating a good assessment of cultural factors relevant to clinical care, which is confirmed by the focus group interview findings, where the clinicians reported the effects of the CFI responses on choice of treatment.

The findings from the focus group interviews are in line with those in the study by Aggarwal and colleagues (2015) on how the CFI affects medical communication. Their results showed increased clinician/patient rapport, with the CFI producing information based on the patient’s story and eliciting the patient’s perception of the illness. Our results also correspond with those of the Mexican study of the CFI (Ramirez Stege & Yarris, 2017), in the sense that clinicians found that it contributed to building trust and clinician-patient rapport, as well as increasing their understanding of the contextual factors influencing mental illness. However, findings from the focus group interviews differ from those of the Mexican study, since the clinicians in our study did not refer to background and identity as being closely related to ethnicity. They made a point of making clear that ethnicity is not the most important factor in the information gathered that was related to background and identity. This may affect the clinicians’ perception of, and the usefulness of, the CFI in the clinical assessment. The clinicians regarded the CFI as also being applicable to patients without an immigrant background.

### Implications for application and future revision of the CFI

Patients and clinicians valued the core CFI. Clinicians praised the person-centred approach that facilitated the patients’ narratives, clarified their context, and contributed to the co-creation of shared understanding. The narrative approach also worked well with interpreters, but sometimes gave rise to difficulties in the translation of abstract wordings. For successful clinical implementation, our findings highlight the importance of teamwork. Even in busy clinical settings, it is important to allow time to listen to the patient’s narrative and to transmit the information gathered by the CFI to all the professionals that are engaged in the psychiatric assessment of the individual patient. This can be a challenge in settings with a shortage of psychiatrists, and where there are clinical models of working with a division of labour between professions, combined with time pressure. Implementation of the CFI requires a context where there is shared concern for the information gathered. If not, there is a risk that the information will be detached from the formal diagnostic categorisation and other aspects of assessment and treatment.

Our study also indicates that, for clinical use, revising some of the questions is beneficial. In particular, the abstract questions about identity and background were difficult for some patients to understand, and it was also difficult for clinicians to use the explanatory text in the CFI. This was of especial concern in interactions with patients with poor language proficiency and when interpreters were used. However, for others, the questions worked well and provided valuable new information. On the basis of experiences in Pune (India), Paralikar, Deshmukh, and Weiss (2020) suggest that assessing cultural identity requires that an interviewer focus on a patient’s self-ascribed identity and not the views of others or the clinician. They also point to the need for further research on the role of cultural identity.

## Conclusions

This study makes a contribution to knowledge about the Clinical Utility, Feasibility and Acceptability of the core CFI in DSM-5. Patients and clinicians participating in the study found that the CFI in DSM-5 was a feasible, acceptable and clinically useful assessment tool. Findings from the focus group interviews suggest that using the CFI at initial contact can contribute to making psychiatric assessment patient-centred by facilitating the patient’s illness narrative. The results reveal difficulties in CFI wording for some patients, indicating the need for adjustments to make the questions more comprehensible. Additionally, arguments are presented for further refinements of the CFI and for clinical evaluations in diverse settings.

## Note

1 MINI (The Mini International Neuropsychiatric Interview), CGI (Clinical Global Impression), web-based self-report screening tools: AUDIT-C (Alcohol Use Disorder Identification Scale), PHQ-9 (Patient Health Questionnaire) and ASRS (Adult ADHD Self-Report Scale).

## Appendix 1

### Interview guide for the focus group interviews

1. Tell me about your experiences of using the CFI.
  - What was valuable about the interview?
  - What was problematic about the interview?
  - What has been difficult to ask?
  - What information has the CFI contributed?
  - Has the interview given new information?
  - Were any themes revealed through the interviews that did not come up in the other assessment interviews?
  - What was the patient’s reaction to the interview?
  - In what way was your relationship with the patient affected by the interview?
  - Have the interviews affected the patients’ commitment to their treatment?
2. In what way did the interview affect your understanding of the patients you met?
  - How has the CFI affected your understanding of the patient’s situation and the problem?
  - How has the CFI affected your understanding of the importance of cultural and contextual factors related to the patient’s illness situation?
3. How has the CFI affected your understanding of the patients’ mental health and need for help?
  - What was useful in the diagnostic situation?
  - Have the interviews affected the diagnostic outcome? How?
  - What was useful for treatment planning?
  - Have the interviews affected treatment interventions? How?
4. What need for improvement and development of the CFI do you see?
  - What aspects did you miss in the interviews?
  - What was difficult or problematic about the CFI?
  - What needs to be improved in the CFI?
5. Finally, some practical questions regarding using the CFI.
  - What was it like to document the interviews? Was there any difference between documenting these interviews and other journal material?
  - How long did the CFI take? Time frame?
